# AI-driven placental biomarkers: a new era for preeclampsia prediction

**DOI:** 10.1097/MS9.0000000000003787

**Published:** 2025-08-28

**Authors:** Raja Haris Shahid, Maliha Khalid, Muhammad Talha, Aminath Waafira

**Affiliations:** a Azad Jammu & Kashmir Medical College Muzzaffarabad AJK; b Jinnah Sindh Medical University, Karachi, Pakistan; c King Edward Medical University, Lahore, Pakistan; d Department of Medicine, The Maldives National University, Malé, Maldives

**Keywords:** artificial intelligence, maternal health, placental biomarkers, preeclampsia prediction

## Abstract

Preeclampsia (PE) remains a major contributor to maternal and perinatal morbidity and mortality worldwide, accounting for tens of thousands of maternal deaths annually. Advances in artificial intelligence (AI) have enabled the development of predictive models incorporating placental biomarkers such as placental growth factor (PlGF), increasing early detection accuracy from 47% with traditional methods to as high as 75% in early gestation. Early identification enables timely interventions – including low-dose aspirin, increased monitoring, and planned delivery – that can significantly reduce adverse outcomes. While biomarker-based AI models require infrastructure investment, their integration into healthcare systems, particularly in resource-limited settings, offers substantial cost-effectiveness and the potential to reduce preventable deaths. Successful implementation depends on accurate data collection, workforce training, standardized biomarker assays, and ongoing model validation across diverse populations. Policymakers, clinicians, and global health stakeholders should prioritize phased adoption strategies, ethical oversight, and public-private collaboration to ensure equitable access and maximize the life-saving potential of AI-based PE prediction tools.

*To the Editor*,

Preeclampsia (PE) is a significant pregnancy-related multisystem condition, affecting approximately 2–5% of pregnancies globally. It stands among the leading contributors to maternal and perinatal illness and death, accounting for an estimated 76 000 maternal and 500 000 infant deaths annually around the world^[1]^. The growing interest in the application of artificial intelligence (AI)-based technologies for the early prediction of preeclampsia via placental biomarkers signifies a dramatic change in maternal health care.

Recent studies have demonstrated that AI algorithms analyzing biomarkers such as placental growth factor (PlGF) and others can predict the risk of developing preeclampsia with up to 75% accuracy in early gestation. After the addition of these placental biomarkers, without these biomarkers, it was only 47% accurate^[2]^. Traditional risk calculators might miss patterns that these models detect from complicated biological interactions. Appropriate therapies that are known to significantly decrease maternal and fetal problems, such as low-dose aspirin medication, more frequent monitoring, or early delivery when necessary, are made possible by this early discovery possible by using AI Algorithms^[3]^. AI models for predicting PE risk can be cost-effective, particularly those developed using existing national health records, thus no new data collection costs. However, models incorporating biomarkers may be more expensive due to the need for specific infrastructure. Their successful clinical application requires accurate recording of maternal characteristics and medical history, proper training for personnel measuring biomarkers, regular audits of results, standardization of biomarkers across diverse populations and assays, consistent outcome measures, and thorough outcome ascertainment^[4]^.

Investing in AI-based PE screening can be a cost-effective strategy to significantly improve maternal health and reduce preventable deaths, especially in resource-limited settings, by enabling early identification of high-risk pregnancies and timely interventions. Policymakers should prioritize funding for integrating these AI models into routine prenatal care, allocating resources for technology, provider training, and continuous validation to ensure quality care and health equity for all pregnant women. Ongoing research efforts should focus on expanding databases and validating the performance of AI in diverse populations, leading to the development of more sophisticated prediction models with improved accuracy^[5]^. Our work is in line with the TITAN Guidelines on the need for transparency in AI use in healthcare^[6]^.

Global health initiatives must prioritize investment in maternal health technologies and adopt a phased implementation strategy, starting in tertiary hospitals and supported by local validation studies, to facilitate equitable access to AI-based PE prediction tools. For clinical integration to be successful, healthcare providers must be trained to interpret AI outputs. However, ethical supervision is necessary to guarantee that these technologies continue to be open, objective, and complementary to clinical judgment rather than a replacement for it. To support biomarker testing and promote public-private partnerships for data-driven maternal care, governments and foreign donors should work together. These predictive tools have the potential to drastically lower avoidable maternal deaths when used promptly, especially in settings with limited resources.

## Data Availability

None.
